# Is There Evidence for the Specificity of Closed-Loop Brain Training in the Treatment of Internalizing Disorders? A Systematic Review

**DOI:** 10.3389/fnins.2022.821136

**Published:** 2022-03-10

**Authors:** Tyson Michael Perez, Jerin Mathew, Paul Glue, Divya B. Adhia, Dirk De Ridder

**Affiliations:** ^1^Department of Surgical Sciences, University of Otago, Dunedin, New Zealand; ^2^Department of Psychological Medicine, University of Otago, Dunedin, New Zealand; ^3^Centre for Health, Activity, and Rehabilitation Research, University of Otago, Dunedin, New Zealand

**Keywords:** EEG, neurofeedback, systematic review, internalizing disorders, emotional disorders, OCD, PTSD, major depressive disorder (MDD)

## Abstract

**Introduction:**

Internalizing disorders (IDs), e.g., major depressive disorder (MDD), posttraumatic stress disorder (PTSD), obsessive-compulsive disorder (OCD) are the most prevalent psychopathologies experienced worldwide. Current first-line therapies (i.e., pharmacotherapy and/or psychotherapy) offer high failure rates, limited accessibility, and substantial side-effects. Electroencephalography (EEG) guided closed-loop brain training, also known as EEG-neurofeedback (EEG-NFB), is believed to be a safe and effective alternative, however, there is much debate in the field regarding the existence of specificity [i.e., clinical effects specific to the modulation of the targeted EEG variable(s)]. This review was undertaken to determine if there is evidence for EEG-NFB specificity in the treatment of IDs.

**Methods:**

We considered only randomized, double-blind, sham-controlled trials. Outcomes of interest included self/parent/teacher reports and clinician ratings of ID-related symptomatology.

**Results:**

Of the four reports (total participant number = 152) meeting our eligibility criteria, three had point estimates suggesting small to moderate effect sizes favoring genuine therapy over sham, however, due to small sample sizes, all 95% confidence intervals (CIs) were wide and spanned the null. The fourth trial had yet to post results as of the submission date of this review. The limited overall number of eligible reports (and participants), large degree of inter-trial heterogeneity, and restricted span of ID populations with published/posted outcome data (i.e., PTSD and OCD) precluded a quantitative synthesis.

**Discussion:**

The current literature suggests that EEG-NFB may induce specific effects in the treatment of some forms of IDs, however, the evidence is very limited. Ultimately, more randomized, double-blind, sham-controlled trials encompassing a wider array of ID populations are needed to determine the existence and, if present, degree of EEG-NFB specificity in the treatment of IDs.

**Systematic Review Registration:**

[https://www.crd.york.ac.uk/prospero], identifier [CRD42020159702].

## Introduction

Internalizing disorders (IDs), e.g., major depressive disorder (MDD), posttraumatic stress disorder (PTSD), and obsessive-compulsive disorder (OCD), are the most prevalent psychopathologies experienced worldwide (Demyttenaere et al., 2004; Kessler et al., 2005, 2007, 2009; Oakley-Browne et al., 2006) and can be broadly characterized by a proclivity to direct distress inwardly (Buchan et al., 2014; Carragher et al., 2015; Krueger and Eaton, 2015; Rhee et al., 2015; Kotov et al., 2017). Although effective for many, traditional frontline ID treatments (i.e., pharmacotherapy and/or psychotherapy) have significant short-comings including substantial long-term failure rates (Haller et al., 2015; James et al., 2015; Peters et al., 2016; Pinter et al., 2019), lack of access (Andrade et al., 2014; Schoenberg and David, 2014; Bandelow and Michaelis, 2015; Haller et al., 2015; Möller et al., 2016) and marked adverse side-effects (Tiller, 2013; Haller et al., 2015; Alvares et al., 2016; Möller et al., 2016; Pinter et al., 2019). Moreover, a decades long drought in the discovery of new compounds has prompted pharmaceutical companies abandon the neuropsychiatric space (Buzsáki and Watson, 2012) leading to appeals from around the world for innovative treatments (Flisher et al., 2007; Haller et al., 2015; Kris, 2018; Pinter et al., 2019).

With aberrations in the brain’s electrical activity well documented in IDs (Pizzagalli et al., 2002; Jokić-Begić and Begić, 2003; Alhaj et al., 2010; Iosifescu, 2011; Buzsáki and Watson, 2012; Wahbeh and Oken, 2013), closed-loop brain training of electrophysiological signals, also known as electroencephalography neurofeedback (EEG-NFB), has been touted as a possible solution. EEG-NFB is non-invasive form of biofeedback that teaches the brain to modify its function *via* a closed-loop brain-computer interface whereby an exogenous sensory stimulus (e.g., audible tone) is fed back to the participant in real-time following some pre-determined electrical activity recorded from the scalp (Collura, 2013; Marzbani et al., 2016; Sitaram et al., 2016; Arns et al., 2017; Orndorff-Plunkett et al., 2017). EEG-NFB is widely believed to work predominantly through a form of associative learning known as operant conditioning whereby the probability of some given (neural) behavior is modified *via* a temporally associated reinforcing stimulus (Enriquez-Geppert et al., 2017; Orndorff-Plunkett et al., 2017; Alkoby et al., 2018). Although the use of EEG-NFB for IDs in routine clinical psychiatric practice has yet to receive widespread support (Begemann et al., 2016; Arns et al., 2017; Omejc et al., 2019), there is substantial evidence that EEG-NFB might be efficacious (Schoenberg and David, 2014; Reiter et al., 2016; Van Der Kolk et al., 2016; Cheon et al., 2017; Noohi et al., 2017; Orndorff-Plunkett et al., 2017; Ros et al., 2017; Panisch and Hai, 2018; Askovic et al., 2019; Bell et al., 2019; Chiba et al., 2019; Wang et al., 2019; Tolin et al., 2020; Hou et al., 2021).

That said, skeptics assert that EEG-NFB’s efficacy derives exclusively from non-specific factors (e.g., expectations, demand characteristics, and context) based primarily on a collection of randomized, sham/placebo-controlled trials for attention deficit hyperactivity disorder (ADHD) which demonstrated comparable clinical improvements in both experimental and sham groups (Thibault et al., 2016, 2018; Schönenberg et al., 2017a,b; Ghaziri and Thibault, 2019; Arnold et al., 2021). EEG-NFB proponents’ most salient objection to this conclusion is that evidence of differential targeted EEG-learning (i.e., greater improvement in the trained electrophysiological variable(s) in genuine vs. sham EEG-NFB groups), considered by many to be essential for a valid evaluation of EEG-NFB’s specificity (Sherlin et al., 2011; Kerson, 2013; Arns et al., 2014; Holtmann et al., 2014; Zuberer et al., 2015; Szewczyk et al., 2018; Witte et al., 2018), was noticeably absent in the trials presented as evidence for wholly non-specific effects (Pigott et al., 2018; Trullinger et al., 2019). Remediation of this apparent shortcoming can be complicated, however, due to a lack of established guidelines for determining successful EEG-learning (Weber et al., 2020).

The aim of our review was to comprehensively evaluate all available randomized, double-blind, sham/placebo-controlled trials in an ID population for evidence of EEG-NFB specificity *via* differences in clinical outcomes (i.e., symptom rating scales) between genuine and sham EEG-NFB groups.

## Materials and Methods

### Registration and Protocol

This review was prospectively registered on the International Prospective Register of Systematic Reviews (PROSPERO) under the registration number: CRD42020159702. The protocol for this systematic review and meta-analysis has been published previously (Perez et al., 2021) and uploaded in PDF format to PROSPERO.

### Eligibility Criteria

We considered all EEG-NFB reports involving humans with at least one ID diagnosis per the Diagnostic and Statistical Manual of Mental Disorders [DSM; (American Psychiatric Association [APA], 2013)] or the International Classification of Diseases [ICD; (World Health Organization [WHO], 2018)] with no exclusion by language, locality, ethnicity, age, or sex. Regarding outcome assessments, all reports were required to include data from at least one clinical rating scale assessing one or more core symptoms of the disorder(s) under investigation. To minimize bias and control for non-specific effects, we included only randomized, double-blind (participants and outcome assessors), sham/placebo-controlled (i.e., feedback contingent on either a random signal, the activity from a different person’s brain, or a signal from the participant’s own brain derived from a region unrelated to the condition under study) reports. Concomitant interventions were permitted provided they were identical for both active and sham groups.

### Information Sources

Studies eligible for review were identified in a literature search from earliest dates within multiple databases (Table 1). Of note, we decided to remove database limit options from our protocol in order to broaden the scope of our searches. Databases were last searched on 23 November 2021. Considering the known importance of including unpublished data in systematic reviews (Trespidi et al., 2011), we also searched various clinical trial registries including ClinicalTrials.gov^1^, the World Health Organization’s International Clinical Trials Registry Platform (ICTRP)^2^, and the Australia New Zealand Clinical Trials Registry (ANZCTR)^3^ to identify completed, unpublished trials. Notably, the ICTRP indexes trial registrations from 17 registries around the world. All registries were last accessed on 23 November 2021. Additionally, reference lists of included articles and relevant systematic reviews were manually screened to identify additional studies.

**TABLE 1:** Platforms/databases and years of coverage.

Platform/Database	Years of coverage
**Ovid**	
AMED (allied and complementary medicine)	1985 to present
CENTRAL (cochrane central register of controlled trials)	1991 to present
MEDLINE and Epub Ahead of Print, In-Process, In-Data-Review and Other Non-Indexed Citations, Daily and Versions	1946 to present
Embase Classic + Embase	1947 to present
APA PsycExtra	1908 to present
APA PsycInfo	1806 to present
Scopus	1788 to present
Pubmed	Late 1700s to present

### Search Strategy

Literature search strategies were developed using medical subject heading (MeSH) and text words related to IDs and neurofeedback. The search strategies were developed by author TMP with guidance from the University of Otago’s Health Sciences librarian. A detailed account of the search strategy for each database and registry can be found in Supplementary Material.

### Selection Process

TMP collated the list of possible records for inclusion and exported them from each database to EndNote (version X9.2) where duplicates were located using EndNote’s duplicate identification strategy (i.e., identifying references in a library of the same reference type with matching Author, Year, and Title fields) and then removed manually. Two independent reviewers (TMP and JM) screened titles and abstracts for eligibility. In cases of disagreement, consensus on articles to assess for eligibility was reached by discussion between TMP and JM. When disagreements couldn’t be resolved, a third team member (DBA) was enlisted to make the final decision. TMP and JM then independently assessed full-text reports appearing to meet the inclusion criteria or when there was any uncertainty. Again, in cases of disagreement, consensus on inclusion/exclusion was reached *via* discussion and, when needed, DBA was consulted to make the final decision. When necessary to resolve questions regarding eligibility, TMP sought additional information from study authors *via* a maximum of three electronic (i.e., email and/or ResearchGate) requests. Notably, two non-English language reports [(Biriukova et al., 2005; Eskandari et al., 2014)] were translated during our full-text eligibility assessments. One report [(Biriukova et al., 2005)] was entered into Google Translate by our team and the subsequent output was validated by the study’s last author. The other (Eskandari et al., 2014) was translated by a native speaker here at the University of Otago. Reasons for excluding trials were recorded. Neither TMP nor JM were blinded to the journal titles, trial authors, or institutions.

### Data Collection Process

Data was extracted by TMP and independently verified by JM. In cases of disagreement, consensus was reached *via* discussion. For all three included reports, trial authors were successfully contacted *via* email for clarification of trial details and/or to obtain missing data.

### Data Items

Our primary outcome of interest was clinician ratings or self/parent/teacher reports of ID-related symptomatology. In trials incorporating multiple domains (e.g., clinician ratings *and* self/parent/teacher reports), a single scale was selected based on a hierarchy (i.e., clinician > self > parent > teacher). When multiple rating scales within a given domain were assessed, the validated scale querying the most core/central feature(s) of the condition under study (as determined by our content expert PG) was selected. In cases where multiple values for a single scale (i.e., total vs. sub-scale scores) were assessed, the total scores were used. Notably, as it has been postulated that longer-term outcomes may help to clarify the issue of specificity (Van Doren et al., 2019), in trials with multiple post-treatment data collection time-points, scores obtained furthest from treatment termination were given preference. To date, standard EEG-NFB protocols have not been established for the treatment of IDs (Banerjee and Argáez, 2017), therefore, no protocols were excluded.

### Study Risk of Bias Assessment

The risk of bias for each eligible report was assessed using the Cochrane Risk of Bias tool version 2 (RoB 2.0) for randomized trials which covers 5 domains (domain 1: risk of bias arising from the randomization process; domain 2: risk of bias due to deviations from the intended interventions; domain 3: risk of bias due to missing outcome data; domain 4: risk of bias in measurement of the outcome; domain 5: risk of bias in the selection of the reported result) (Sterne et al., 2019). Two reviewers (TMP and JM) independently applied the tool to each trial and recorded supporting information/justifications for risk of bias judgments (low, some concerns, high) in each domain. Further, an overall summary risk of bias judgment (low, some concerns, high) was made for each report determined by the highest risk of bias level across all 5 domains. All decisions were guided by the published criteria for judging the risk of bias (Higgins et al., 2021). If there was insufficient detail reported in the study, the original study investigators were contacted *via* email for more information. Disagreements were resolved in discussion and, in cases where consensus was not reached, a third team member (DBA) was enlisted to make the final decision.

### Effect Measures

Because the included trials utilized different measurement scales to assess clinical outcomes, standardized mean differences (SMDs) and 95% confidence intervals (CIs) were calculated using Hedges’ adjusted g in RevMan (version 5.4.1) and presented in a forest plot. In our forest plot, an SMD of 0 is represented by a black vertical line with negative (left-sided) and positive (right-sided) values favoring sham and active groups, respectively. Each trial’s SMD point estimate and 95% CI are represented by a green vertical dashes and bilateral black horizontal lines, respectively. The SMD expresses the size of the intervention effect relative to the variability observed under the assumption that between-study standard deviation variations reflect differences in measurement scales rather than variability in the study populations or reliability in outcome measures (Deeks et al., 2021). SMDs of 0.2, 0.5, and 0.8 are generally interpreted to reflect small, medium, and large effects sizes, respectively (Cohen, 1988).

### Synthesis Methods

The limited overall number of eligible reports (and participants), large degree of inter-trial heterogeneity, and restricted span of ID populations with published/posted outcome data (i.e., PTSD and OCD) precluded a robust quantitative synthesis investigating the potential for EEG-NFB specificity in the treatment of IDs.

### Reporting Bias Assessment

To assess for reporting biases (e.g., selective non-publication and selective non-reporting of results), two independent reviewers (TMP and JM) searched various registries for unpublished trials as well as to compare published trial report outcomes to outcomes specified in their registrations to help guide ratings for domain 5 (i.e., risk of bias in the selection of the reported result) of the RoB 2.0. When registrations were unavailable, we compared the congruency of the section “Materials and Methods” and section “Results” for each trial. Disagreements were resolved in discussion and, when necessary, DBA was consulted to make the final decision.

### Certainty Assessment

Two independent reviewers (TMP and JM) assessed the certainty in the body of the evidence as it related to the trials that contributed data to the meta-analysis using the Grading of Recommendations, Assessment, Development and Evaluations (GRADE). A certainty ranking of high (there is a lot of confidence that the true effect lies close to that of the estimated effect), moderate (the true effect is probably close to the estimated effect), low (the true effect might be markedly different from the estimated effect.), or very low (the true effect is likely to be substantially different from the estimated effect) was assigned by the software based on the reviewers assignments across a number of domains including study design (i.e., randomized trial or observational study), risk of bias (not serious, serious, or very serious), inconsistency (not serious, serious, or very serious), indirectness (not serious, serious, or very serious), imprecision (not serious, serious, or very serious). We used the methods and recommendation described in the GRADE handbook (Schünemann and Oxman, 2013). Disagreements were resolved in discussion and, when necessary, DBA was consulted to make the final decision. A GRADE profile (v2) was generated using GRADEpro GDT software (Evidence Prime, 2020). Where necessary, we provided explanations for our assignments using footnotes to aid the reader’s understanding of the results.

## Results

### Study Selection

**FIGURE 1:**
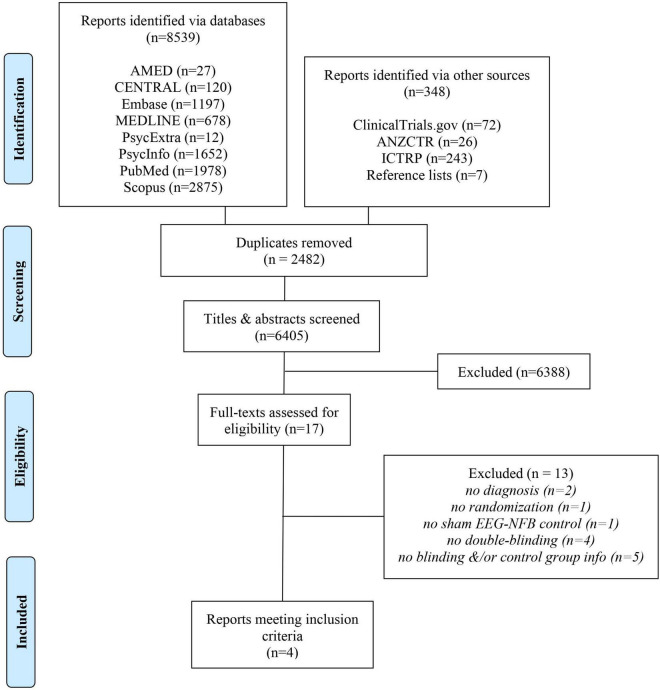
Flow of screening and selection of studies.

Our search uncovered 8,887 records in databases, registries, and relevant reference lists (Figure 1). After duplicates were removed, we screened 6,405 records, from which we reviewed 17 full-text documents. Ultimately, four reports met our eligibility criteria.

Following full-text assessments, we excluded 13 studies from our review (Watson and Herder, 1980; Rice et al., 1993; Biriukova et al., 2005; Iranian Registry of Clinical Trials, 2008; Choi et al., 2011; Eskandari et al., 2014; Sadjadi and Hashemian, 2014; Hashemian and Sadjadi, 2015; Mennella et al., 2017; Noohi et al., 2017; Viereck et al., 2017; Esmaeeli, 2021; Hou et al., 2021) for various reasons (Figure 1) which included a lack of randomization (Watson and Herder, 1980), double-blinding ((Rice et al., 1993; Biriukova et al., 2005; Iranian Registry of Clinical Trials, 2008; Hou et al., 2021), sham EEG-NFB controls (Choi et al., 2011), ID diagnosis (Mennella et al., 2017) and (Viereck et al., 2017), or missing details regarding the control group and/or blinding (Eskandari et al., 2014; Sadjadi and Hashemian, 2014; Hashemian and Sadjadi, 2015; Noohi et al., 2017; Esmaeeli, 2021). With respect to these latter five trials excluded due to missing information, one failed to provide the trialist’s contact information (Esmaeeli, 2021), whereas, for the other four, we made multiple attempts to contact the authors *via* email and/or ResearchGate, however, they failed to respond to our requests.

### Study Characteristics

An detailed overview of the eligible trials (ClinicalTrials.gov, 2000a; Nederlands Trial Register, 2004; Kopřivová et al., 2013; Nicholson et al., 2020) is presented in Table 2. As can be seen, there is significant clinical (e.g., population) and methodological (e.g., clinical outcome scale) heterogeneity across trials.

**TABLE 2:** Summary of EEG-neurofeedback studies included in this review.

Study	Diagnosis (population)	Genuine: -Sample size (drop-outs) -Age range (mean) -Males/females	Sham -Sample size (drop-outs) -Age range (mean) -Males/females -Sham type	Intervention -Target(s)/goal(s) -Feedback modality -Dose/frequency/duration -individualized/standardized training -% positive feedback -thresholding type	Scale	EEG-Learning	Follow-up
Kopřivová et al. (2013)	OCD (hospitalized in-patients)	-*n* = 8 (2) -19–42 (27) -1 male/7 females	-*n* = 10 (0) -19–42 (28.7) -3 males/7 females -replay	- 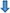 ” (3–8 Hz) or low beta (13–16 Hz) in abnormal IC -Visual + auditory -25 30-min sessions/”every working day”/6 wks -individualized -∼25% -Automatic	Y-BOCS	Active > Sham	Immediate
Onton 2016 (ClinicalTrials.gov, 2000a)	PTSD (active military)	-*n* = 24 (12) -18–40 (30.4) -22 males/2 females	-*n* = 24 (8) -18–40 (29.4) -21 males/3 females -replay	-Infra-low (0.0001 Hz) at T4/P4 or T3/T4 -Visual + tactile + auditory -16 30-min sessions/4x wk/4 wks -Standardized -N/A -N/A	ALI	Not assessed	Immediate
Nicholson et al. (2020)	PTSD (community sample)	-*n* = 18 (0) -21–59 (40.3) -6 males/12 females	-*n* = 18 (0) -21–59 (46.3) -4 males/14 females -Replay	- 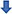 Alpha (8–12 Hz) at Pz -Visual -20 20-min sessions/1× wk/20 wks -Standardized -∼65% -Manual	CAPS	Active > Sham	3 months
Peters 2017 (Nederlands Trial Register, 2004)	MDD (community sample)	-*n* = 25 (NR) -18–65 (NR) -NR	-*n* = 25 (NR) -18–65 (NR) -NR -Random signal	- 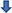 Alpha (7.8–13.1 Hz) asymmetry at F3/F4 -Visual -18 sessions/3x wk/6 wks -Standardized -N/A -N/A	QIDS-SR	NR	*3 months

OCD, obsessive-compulsive disorder; PTSD, post-traumatic stress disorder; MDD, major depressive disorder; IC, independent component; Y-BOCS, Yale-Brown Obsessive-Compulsive Scale; ALI, anxiety level index; CAPS, clinician administered PTSD scale; QIDS-SR, quick inventory of depressive symptomatology – self report; NR, not reported; N/A, not applicable; *, not performed.

### Risk of Bias in Studies

**FIGURE 2:**
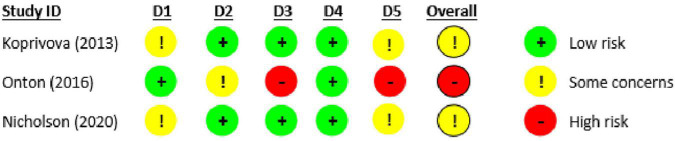
Risk of bias in eligible studies with published/posted outcome data using version 2 of the Cochrane risk-of-bias tool for randomized trials (RoB 2.0).

We used the RoB 2.0 to assess risk of bias for the eligible trials *with* posted/published outcome data (Figure 2). The overall risk of bias was deemed moderate (i.e., “some concerns”) for two of the trials (Kopřivová et al., 2013; Nicholson et al., 2020) and high for the third (ClinicalTrials.gov, 2000a) suggesting some methodological heterogeneity across trials. Concerns included a lack of pre-registered protocols and/or analysis plans with which to compare the final reports and outcomes.

### Results of Individual Studies

**FIGURE 3:**
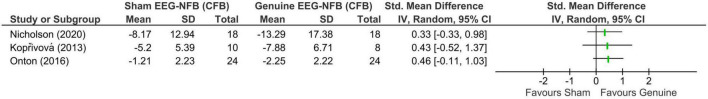
Forest plot showing standardized mean differences in change-from-baseline scores between sham and genuine EEG-neurofeedback using a Hedges’ (adjusted) g.

Figure 3 displays summary statistics [CFB mean and SD, sample size (Total)] for the sham and genuine EEG-NFB groups along with the SMD and 95% CI of the continuous outcome (reduction in internalizing symptoms) for each study. Of note, one eligible trial [i.e., (Nederlands Trial Register, 2004)] is absent due to a failure to post/publish results. Unfortunately, the lead trialist was unwilling to share unpublished data. For all trials with final results, trialists were successfully contacted *via* email for additional data not provided in the original report. Baseline outcome measures were reported to be equivalent between genuine and sham groups in the two published studies (Kopřivová et al., 2013; Nicholson et al., 2020) and were not reported in the unpublished trial (ClinicalTrials.gov, 2000a). Across trials, point estimates consistently demonstrated small to moderate effect sizes favoring genuine over sham EEG-NFB, however, in all cases 95% CIs were relatively wide and spanned the null line. Specifically, SMDs [95% CIs] were 0.42 [−0.52, 1.37] (Kopřivová et al., 2013), 0.46 [−0.11, 1.03] (ClinicalTrials.gov, 2000a), and 0.33 [−0.33, 0.99] (Nicholson et al., 2020). The two published trials [i.e., (Nicholson et al., 2020) and (Kopřivová et al., 2013)] did assess for differential targeted EEG-learning with group point estimates suggesting a trend toward greater improvements (i.e., reductions) in the trained EEG variable(s) in the genuine EEG-NFB groups relative to sham, however, correlation analyses between changes in the targeted EEG variable(s) and clinical outcome measures were not performed.

### Reporting Biases

Overall, we feel the risk of *selective outcome reporting* was low as all trials’ methods and results sections were congruent and well-accepted scales were generally incorporated for the conditions under study, however, there remains a risk of *selective analysis reporting* due to a lack of pre-specified analysis plans per trial registry records or published protocols. Additionally, we have concerns surrounding *publication bias* as we were unable to include the outcome data from one of the completed trials meeting our eligibility criteria [i.e., (Nederlands Trial Register, 2004)] because they have yet to be made publicly available despite being “terminated [in 2017] because of disappointing mid trial results” (personal communication with the lead trialist, 7 July 2020).

### Certainty of Evidence

As seen in the GRADE profile (Table 3), although the certainty of the evidence is initially rated “high” due to the incorporation of the three RCTs with published/posted outcomes, it was subsequently downgraded three steps to “very low” due to (1) “serious” concerns surrounding the cumulative risk of bias, (2) “serious” concerns regarding the imprecision (i.e., wide 95% CIs), and (3) “strongly suspected” publication bias (i.e., no published/posted outcomes from the fourth eligible trial).

**TABLE 3:** GRADE certainty of evidence table.

Certainty assessment						

No. of participants (studies)	Risk of bias	Inconsistency	Indirectness	Imprecision	Publication bias	Overall certainty of evidence
102 (3)	Serious	Not serious	Not serious	Serious	Publication bias strongly suspected	⊕○○○ VERY LOW

## Discussion

To our knowledge, this is the first systematic review exploring the potential for EEG-NFB specificity (i.e., specific effects) in ID populations. Of the four eligible reports identified, three (2 PTSD and 1 OCD) trials provided outcome data with point estimates suggesting EEG-NFB specificity, however, effect sizes were modest with wide 95% CIs spanning the null. The fourth trial, which recruited people with MDD, has yet to publish its results although, according to the lead trialist, the results were “disappointing.” Notably, it could be argued that the protocols undertaken in the eligible trials may have mitigated between-group differences and the overall effect size. More specifically, it has been reported that individualized training is typical in clinical settings and may lead to augmented effects and better outcomes (Hammond, 2010; Omejc et al., 2019; Weber et al., 2020), however, only one of the trials utilized an individualized training paradigm insofar as the targeted frequency or frequency band was based on individual deviations from a normative sample. Furthermore, it has been postulated that non-specific (i.e., placebo) effects are transient, therefore longer term follow-ups serve to better elucidate specificity (Van Doren et al., 2019) yet only one of the eligible trials incorporated any follow-up (i.e., 3 months). Although highly speculative, against this general backdrop of standardized protocols and lack of extended follow ups, theoretically the effect sizes may have been downwardly biased.

Some limitations of the current review should be noted. Firstly, although we had no exclusions by language, we may have unintentionally overlooked some non-English language trials meeting our criteria considering the fact that *coverage bias* (i.e., systematic exclusion of journals from certain countries and/or in certain languages) has been reported in some of the databases that were utilized (Pilkington et al., 2005). That said, the exclusion of non-English language publications from quantitative reviews of clinical interventions has been shown to have little-to-no effect on results and conclusions (Nussbaumer-Streit et al., 2020). Secondly, although two of the trials did assess for differential group EEG-learning (i.e., reduction in the power of the targeted frequency band) with point estimates in both suggesting greater improvements in the genuine group relative to sham, neither correlated this learning with clinical outcomes. Thirdly, outcome data were only available for PTSD and OCD populations, thereby excluding information on the most prevalent IDs (i.e., anxiety and depressive disorders) and limiting the generalizability of our findings. Lastly, due to the small number and extreme heterogeneity of eligible trials, a proper meta-analysis could not be undertaken. A major potential contributor to these latter two limitations was that many candidate trials failed to provide sufficient details necessitating requests for additional information and, in many cases, exclusion of their data due to non-responsive trialists. Reporting issues in RCTs are widespread (Moher et al., 2012) which have prompted skeptics and proponents alike to come together to publish a consensus paper on the reporting and design of neurofeedback studies (Ros et al., 2020). It is our hope that these guidelines will be widely adopted in the field and incorporated into future publications. Additionally, the lack of eligible reports, small participant numbers, and lack of replication may be explained, in part, by the significant investment in resources (i.e., time, human, and monetary) required to undertake EEG-NFB trials of this nature although this too is highly speculative.

Neuropsychiatric disorders are among the most common causes of morbidity and mortality (Kessler et al., 2009) with rates markedly increasing world-wide in recent years (Duffy et al., 2019; Keyes et al., 2019; Twenge et al., 2019; Haidt and Allen, 2020; Pfeifer and Allen, 2021). Among them, IDs, which are characterized by distress experienced inwardly (Cosgrove et al., 2011; Buchan et al., 2014), are the most prevalent. Recently, a government inquiry here in Aotearoa New Zealand has shed light on the shortcomings of traditional front-line treatments (i.e., pharmacotherapy and/or psychotherapy) and urged wider implementation of alternative approaches to the treatment of mental health problems (Kris, 2018). Likewise, scientists abroad have called for more research into “novel interventions that may be based on altering plasticity or returning circuitry rather than neurotransmitter pharmacology” (Insel and Wang, 2010). EEG-NFB is a non-invasive treatment that appears to be generally safe and efficacious when directed by a qualified practitioner (Hammond and Kirk, 2008), however, despite over 50 years since its inception, questions regarding the nature of those effects abound. Specifically, there is much controversy surrounding the existence of EEG-NFB specificity [i.e., specific effects deriving from modulation of the EEG variable(s) of interest]. The current systematic review suggests that genuine EEG-NFB may induce specific effects in at least some ID populations (i.e., PTSD and OCD). That said, the evidence is very limited, thereby warranting more randomized, double-blind, sham-controlled studies to verify the existence and, if present, degree of specificity across the spectrum of IDs. Encouragingly, we are aware of a number of registered randomized, double-blind, sham-controlled EEG-NFB trials in ID populations [i.e., (ClinicalTrials.gov, 2000b; Australian New Zealand Clinical Trials Registry, 2005a,b)] which, when incorporated into a future meta-analysis, may help bring clarity to this intriguing and important topic.

## Data Availability Statement

The original contributions presented in the study are included in the article/Supplementary Material, further inquiries can be directed to the corresponding author.

## Author Contributions

TP is the guarantor and drafter of the manuscript and developed and implemented the search strategies. PG provided expertise on mental health disorders. JM assisted with the screening, data extraction, and assessments. All authors contributed to the development of the selection criteria, the risk of bias assessment strategy and data extraction criteria with guidance from the Health Sciences Librarian and read, provided feedback, and approved the final manuscript.

## Conflict of Interest

The authors declare that the research was conducted in the absence of any commercial or financial relationships that could be construed as a potential conflict of interest. The reviewer PN declared a past co-authorship with several of the authors DA and DD to the handling editor.

## Publisher’s Note

All claims expressed in this article are solely those of the authors and do not necessarily represent those of their affiliated organizations, or those of the publisher, the editors and the reviewers. Any product that may be evaluated in this article, or claim that may be made by its manufacturer, is not guaranteed or endorsed by the publisher.

## Footnotes

1
www.clinicaltrials.gov

2
www.who.int/clinical-trials-registry-platform

3
www.anzctr.org.au

## Abbreviations

ALI: anxiety level index
CAPS: Clinician Administered PTSD Scale
CFB: change-from-baseline
EEG-NFB: electroencephalography neurofeedback
GRADE: grading of recommendationsassessmentdevelopment and evaluations
IC: independent component
ICTRP: World Health Organization’s International Clinical Trials Registry Platform
ANZCTR: Australia New Zealand Clinical Trials Registry
IDs: internalizing disorders
MDD: major depressive disorder
OCD: obsessive-compulsive disorder
PI: post-intervention
PROSPERO: international prospective register of systematic reviews
PTSD: post-traumatic stress disorder
RoB 2.0: Cochrane Risk of Bias tool version 2
SMD: standardized mean difference
Y-BOCS: Yale-Brown Obsessive Compulsive Scale.
