# Supplementary material for: Is There Evidence for the Specificity of Closed-Loop Brain Training in the Treatment of Internalizing Disorders? A Systematic Review
Source: Front Neurosci. 2022 Mar 10;16:821136. doi: 10.3389/fnins.2022.821136 (PMC8960197; doi:10.3389/fnins.2022.821136)
Supplement: Supplementary file 1 [file Table_1.DOCX]

**SEARCH STRATEGIES**

**AMED/Ovid (advanced search)**

exp depression/ OR anxiety/ OR exp fear/ OR exp depressive disorder/ OR exp mood disorders/ OR exp anxiety disorders/ OR exp neurotic disorders/ OR exp anorexia/ OR exp eating disorders/

AND

exp biofeedback/

**Cochrane Central Register of Controlled Trials/Ovid (advanced search)**

exp depression/ OR exp anxiety/ OR exp fear/ OR exp anxiety disorders/ OR exp mood disorders/ OR exp neurotic disorders/ OR exp anorexia/ OR exp eating disorders/

AND

exp Biofeedback, Psychology/ OR exp Brain-Computer Interfaces/

**Embase/Ovid (advanced search)**

exp fear/ OR exp anxiety disorder/ OR exp emotional disorder/ OR exp neurosis/ OR exp mood disorder/ OR exp feeding disorder/ OR exp appetite disorder/ OR exp eating disorder/

AND

exp biofeedback/ OR exp brain computer interface/

**MEDLINE/Ovid (advanced search)**

exp depression/ OR exp anxiety/ OR exp fear/ OR exp anxiety disorders/ OR exp mood disorders/ OR exp neurotic disorders/ OR exp anorexia/ OR exp "feeding and eating disorders"/ OR exp "Trauma and Stressor Related Disorders"/

AND

exp Biofeedback, Psychology/ OR exp Brain-Computer Interfaces/

**PsycExtra/Ovid (advanced search)**

exp anxiety/ OR exp anxiety disorders/ OR exp fear/ OR exp internalizing symptoms/ OR exp internalization/ OR exp neurosis/ OR exp affective disorders/ OR exp "Depression (Emotion)"/ OR exp posttraumatic stress disorder/ OR exp "stress and trauma related disorders"/ OR exp eating disorders/

AND

exp biofeedback/ OR exp human computer interaction/

**PsycInfo/Ovid (advanced search)**

exp anxiety/ OR exp anxiety disorders/ OR exp fear/ OR exp internalizing symptoms/ OR exp internalization/ OR exp neurosis/ OR exp affective disorders/ OR exp "Depression (Emotion)"/ OR exp posttraumatic stress disorder/ OR exp "stress and trauma related disorders"/ OR exp eating disorders/

AND

exp biofeedback OR exp Human Computer Interaction/

**PubMed (advanced search)**

((obsessive compulsive OR obsessive-compulsive OR OCD OR anxiety OR depressive OR depression OR fear OR panic OR phobia OR phobic OR neurotic OR neuroses OR neurosis OR emotional disorder OR affective disorder OR mood disorder OR internalizing OR ptsd OR posttraumatic OR post-traumatic OR anorexia OR bulimia OR binge-eating disorder OR binge eating disorder)) AND (biofeedback OR neurofeedback OR neurotherapy OR brain-computer interface OR closed-loop brain training)

**Scopus (advanced search)**

( ( TITLE-ABS-KEY ( "obsessive compulsive" OR "obsessive-compulsive" OR ocd OR anxiety OR depression OR depressive OR fear OR panic OR phobia OR phobic OR neurotic OR neuroses OR neurosis OR "emotional disorder*" OR "affective disorder*" OR "mood disorder*" OR internalising OR ptsd OR posttraumatic OR post-traumatic OR anorexia OR bulimia OR "binge eating disorder" OR "binge-eating disorder" ) ) AND ( TITLE-ABS-KEY ( biofeedback OR neurofeedback OR neurotherapy OR "brain-computer interface" OR "closed-loop brain training" ) ) )

**ICTRP (advanced search)**[**https://apps.who.int/trialsearch/AdvSearch.aspx**](https://apps.who.int/trialsearch/AdvSearch.aspx)

Emotional disorder OR Affective Disorder OR Internalizing OR Anxiety OR Depression OR PTSD OR Phobia OR Phobic OR Panic OR Mood disorder OR OCD OR anorexia OR bulimia OR binge eating disorder (in the Condition)

AND

biofeedback OR neurofeedback OR neurotherapy OR closed-loop brain training OR brain-computer interface (in the Intervention)

Recruitment status is ALL

**ClinicalTrials.gov (advanced search)** [**https://clinicaltrials.gov/ct2/search/advanced?cond=&term=&cntry=&state=&city=&dist=**](https://clinicaltrials.gov/ct2/search/advanced?cond=&term=&cntry=&state=&city=&dist=)

Condition or disease: Emotional disorder OR Affective Disorder OR Anxiety OR Depressive OR Depression OR PTSD OR Post-traumatic OR Posttraumatic OR Phobia OR Phobic OR Panic OR Mood disorder OR obsessive-compulsive OR obsessive compulsive OR OCD OR anorexia OR bulimia

Intervention/treatment: Biofeedback OR Neurofeedback OR Neurotherapy OR Closed-loop brain training OR brain-computer interface

Recruitment status: Completed or Unknown status

**ANZCTR (advanced search)**[**http://www.anzctr.org.au/TrialSearch.aspx**](http://www.anzctr.org.au/TrialSearch.aspx)

Search 1:

Registry: All
Description of Intervention(s)/exposure: Biofeedback
Study type: Interventional
Recruitment status: Completed

Search 2:

Registry: All
Description of Intervention(s)/exposure: Neurofeedback
Study type: Interventional
Recruitment status: Completed

Search 3:

Registry: All
Description of Intervention(s)/exposure: Neurotherapy
Study type: Interventional
Recruitment status: Completed

Search 4:

Registry: All
Description of Intervention(s)/exposure: Closed-loop brain training
Study type: Interventional
Recruitment status: Completed

Search 5:

Registry: All
Description of Intervention(s)/exposure: Brain-computer interface
Study type: Interventional
Recruitment status: Completed
